# SARS-CoV-2 cell receptor gene ACE2 -mediated immunomodulation in breast cancer subtypes

**DOI:** 10.1016/j.bbrep.2020.100844

**Published:** 2020-11-05

**Authors:** Vikas Kumar Bhari, Durgesh Kumar, Surendra Kumar, Rajeev Mishra

**Affiliations:** a Department of Biosciences, Manipal University Jaipur, Rajasthan, India; b Department of Physiology, Government Medical College, Kannauj, Uttar Pradesh, India; c Department of Neurology, Rajendra Institute of Medical Sciences, Ranchi, Jharkhand, India

**Keywords:** COVID-19, ACE2, Breast cancer, Immunomodulation, Genomics

## Abstract

The recent outbreak of severe acute respiratory syndrome coronavirus 2 (SARS-CoV-2) infection has impacted the world severely. The binding of the SARS-CoV-2 virus to the angiotensin-converting enzyme 2 (ACE2) and its intake by the host cell is a necessary step for infection. ACE2 has garnered widespread therapeutic possibility as it is entry/interactive point for SARS-CoV-2, responsible for coronavirus disease 2019 (COVID-19) pandemic and providing a critical regulator for immune modulation in various disease. Patients with suffering from cancer always being on the verge of being immune compromised therefore gaining knowledge about how SARS-CoV-2 viruses affecting immune cells in human cancers will provides us new opportunities for preventing or treating virus-associated cancers. Despite COVID-19 pandemic got center stage at present time, however very little research being explores, which increase our knowledge in context with how SARS-CoV-2 infection affect cancer a cellular level. Therefore, in light of the ACE-2 as an important contributor of COVID-19 global, we analyzed correlation between ACE2 and tumor immune infiltration (TIL) level and the type markers of immune cells were investigated in breast cancer subtypes by using TIMER database. Our findings shed light on the immunomodulatory role of ACE2 in the luminal A subtype which may play crucial role in imparting therapeutic resistance in this cancer subtype.

## Highlights

• The recent outbreak of Severe acute respiratory syndrome coronavirus 2 (SARS-CoV-2) infection has impacted the world severely.
• The binding of the SARS-CoV-2 virus to the angiotensin-converting enzyme 2 (ACE2) and its intake of this complex by the host cell is a necessary step for infection.
• We reported that the differential gene expression of ACE2 defines its pathological role in each molecular subtype of breast cancer differently.
• Our analysis suggested that the expression of ACE2 gene may relate to immune function in luminal A subtype of breast cancer.
• This study is much significant in context with current pandemic in which ACE2 inhibitors are widely used for the COVD-19 treatment.

## 1 Introduction

Prediction of clinical behaviors and patient outcomes in breast cancer is very difficult and its heterogeneity makes it more complicated [1]. The heterogeneous nature of breast cancer is reflected by different staging systems and histopathologic classification [2]. Gene expression profiling is the major criterion for intrinsic classification molecular subtypes of breast cancer known as luminal A, luminal B, HER2-enriched and basal-like highlighting the importance of different treatment responses, therefore, advocating for different therapeutic strategies [1,3,4]. Immune cells are inseparable components of the tumor microenvironment of breast cancer and are reported to be associated with prognosis and survival [[5], [6], [7]]. Tumor-infiltrating immune cells (TIC) has been correlated with better outcome and response to adjuvant therapies in breast cancer [8]. The immune components are identified to correlate with the prognosis of breast cancer and its treatment strategy [9]. However, there is no proof that a single immune cell will play the same role among all four subtypes [10], which makes it mandatory to evaluate each breast cancer subtype.

The unexpected arrival of the Severe acute respiratory syndrome coronavirus 2 (SARS-CoV-2) has broadly and badly impacted all aspects of life including cancer patients. This disease has rapidly spread to at least 235 countries, with nearly 41 million confirmed cases resulting in nearly 1,128,325 confirmed deaths as of October 22, 2020 (https://www.who.int/emergencies/diseases/novel-coronavirus-2019). The coronavirus receptor angiotensin-converting enzyme 2 (ACE2) has been recognized as an entry and interaction receptor (entry gate) for the SARS-CoV-2 virus [11], therefore, ACE2 has been accountable for the pathogenesis for COVID-19. Ever since the transmission of SARS-CoV2 emerged in China, receptor-mediated entry of SARS-CoV2 has been studied extensively [12,13]. The RBD of the spike protein of SARS-CoV2 is responsible for the recognition of the ACE2 receptor and TMPRSS2, a serine protease is employed for S Protein priming [[14], [15], [16]].

COVID-19 severity and progression have been linked not only with the viral load but other associate factors such as age, sex, ethnicity, and several comorbidities with associated disease including cancer [17,18]. Despite its distribution throughout the human tissues, it has been shown that ACE2-expressing organs do not behave in a regular pattern which is one of the matter of deep concern for different clinical COVID-19 disease outcomes [19], suggesting more research is needed to widen therapeutic window. The ACE2 is widely ubiquitously distributed and highly expressed in various tissue such as heart, kidney, brain, testis, and cardiovascular‐relevant tissues [20]. Being highly expressed in these tissues, ACE2 plays a major role in modulation of blood pressure, renal and cardiac functions, therefore its receptor alteration led major cardiac and renal pathophysiology associated disease [21]. Renin-angiotensin-aldosterone system (RAAS) inhibitors, which include angiotensin-converting enzyme (ACE) inhibitors (ACEIs) and angiotensin II receptor type 1 blockers (ARBs) are widely used for treatment of hypertension, heart failure and coronary heart disease. Among all these diseases the major concern is associated with cardiovascular disease which is clinical managed by RAAS inhibitors such as ACEIs and ARBs [22,23]. One of the primary concerns is that use of ACEIs and ARBs, increases the surface expression of ACE2 which will ultimately led to increased susceptibility towards COVID-19 infection as this will facilitate entry of SARS-CoV-2. On the contrary one retrospective multi-center study showed that lower mortality risk among the COVID-19 patients comorbid with hypertension treated with ACEIs and ARBs, warranting further research needed in this field [24].

Recently it has been reported that cancer patients might be more susceptible to severe infection [25], however, this scenario might be contradicted as ACE2 expression varies in different cancer types [26]. Analysis of ACE2 expression levels in different tumor cells not only helps in understanding us about the susceptibility of COVID-19 but also provides useful information about the possible consequence of ACEIs in driving/preventing tumor growth. Given this situation, we use publically available cancer genomics datasets [27] to understand the prevalence of ACE2 receptor in different types of cancer patients with COVID-19, which can be valuable in specific targeting and support the ongoing fight with SARS-CoV-2.

In this study, we first compare the expression of ACE2 in normal, breast cancer patients, and in different stages by using UACLAN and UCSC Xena browser database. After analyzing expression, the relationship between ACE2 and TIL levels in different breast cancer subtypes was investigated using the Tumor Immune Estimation Resource (TIMER) database. Different types of immune cell markers and cells were evaluated in all the subtypes of breast cancer. Our work identified crucial involvement of ACE2 in immunomodulation in luminal A subtype and predict the functional consequence of using ACEIs in this breast cancer subtype, further strengthening the notion that when cancer care and COVID-19 collide, then there is no easy and universal solution to oncologic care.

## 2 Materials and methods

### 2.1 UCSC Xena cancer genomics browser analysis

*University of California Santa Cruz (*UCSC) Xena browser (https://xenabrowser.net) was used to compare the mRNA expression of ACE2 between breast cancer and normal patients and ACE2 expression among breast cancer subtypes [28]. TCGA TARGET GTEx study (n = 1278) was selected to compare ACE2 expression in tumor samples vs. normal samples. After selecting this study, we used a filter option to narrow down the samples associated only with TCGA breast invasive carcinoma and GTEx breast. RSEM norm count dataset was used for the analysis and values were downloaded as log_2_ (norm_count +1). To investigate the ACE2 in breast cancer subtypes, TCGA Breast Cancer (BRCA) (n = 1247) was used. IlluminaHiSeq dataset was used for the analysis and values were download as log_2_ (norm_count +1).

### 2.2 UALCAN analysis

UALCAN database was used to visualize the transcript per million of ACE2 based on breast cancer individual stage. The statistical significance table was provided by the website (http://ualcan.path.uab.edu/analysis.html). [29].

### 2.3 TIMER 2.0 analysis

TIMER 2.0 was used to investigate the ACE2 TIL in breast cancer and its subtypes. It is a comprehensive database that can illustrate the immunomodulation across various cancer types (http://timer.cistrome.org) [30,31]. We evaluated the correlation of ACE2 with TIL (NK cells, Dendritic cells, Neutrophils, and T-cell regulatory) in breast cancer subtypes. After this, the correlation of ACE2 with the immune cell type markers of TLRs, macrophage, monocytes, dendritic cells, neutrophils, and NK cells in breast cancer subtypes was confirmed. Tumor purity was used for *P*-value correction in the case of immune cell correlation analysis. According to correlation values, the heat map was generated by using a web application for comprehensive metabolomics data analysis and interpretation, MetaboAnalyst (http://www.metaboanalyst.ca) [32].

### 2.4 Statistical analysis

Data integration and statistical analysis were performed using GraphPad Prism 8.4.3. Box plots were generated for group comparison. A comparison between two groups was performed using Unpaired *t*-test with Welch's correction. Significance was determined by the *P-*value provided by the database. Spearman correlation values indicated in the tables (Table 1, Table 2, Table 3, Table 4) were provided by the database. *P < 0.05; ***P < 0.001; ****P < 0.0001, was considered statistically significant.

**Table 1:** The ACE2 correlated immune cell markers of toll-like receptors, macrophages, dendritic cells, natural killer cells, neutrophils, monocytes and cytokines in Luminal A subtype of breast cancer.

Cell Type	Gene markers	COR	P-values
*Toll-like receptors*	TLR4	0.264	1.69E-10
	TLR7	0.104	1.34E-02
	TLR8	0.25	1.57E-09
	TLR9	0.35	9.04E-18
*Macrophage*	MS4A4A	0.204	9.85E-07
	CD68	0.084	4.45E-02
	CD163	0.158	1.54E-04
*Dendritic Cells (DCs)*	ITGAX	0.145	5.08E-04
	FSCN1	0.176	2.59E-05
	ADAM19	0.178	2.03E-05
*Natural Killer cells (NK)*	NCR1	0.198	1.92E-06
	NCAM1	0.344	3.44E-17
	KLRB1	0.324	2.63E-15
*Neutrophils*	CEACAM3	0.178	1.96E-05
	FCGR3B	0.115	6.01E-03
	FPR1	0.136	1.21E-03
*Monocyte*	CSF1R	0.15	3.44E-04
	CD86	0.121	3.77E-03
	C3AR1	0.084	4.59E-02
*Cytokines*	IL2	0.203	1.03E-06
	IL1A	0.068	0.101
	IL10	0.200	1.47E-06
	IL19	0.0350	0.403
	IL20	0.006	0.868
	IL21	0.149	0.0003
	TNF	0.057	0.173
	CXCR2	0.166	6.45E-05
	CSF1	0.075	0.071
	CD274	0.251	1.21E-09
	CTLA4	0.075	0.071
	PDCD1	0.250	1.45E-09

**Table 2:** The ACE2 correlated immune cell markers of toll-like receptors, macrophages, dendritic cells, natural killer cells, neutrophils, monocytes and cytokines in Basal-like subtype of breast cancer.

*Cell Type*	Gene markers	COR	P-Values
*Toll-like receptors*	TLR4	0.126	0.081
	TLR7	0.198	0.005
	TLR8	0.140	0.053
	TLR9	0.135	0.060
*Macrophage*	MS4A4A	0.201	0.005
	CD68	0.070	0.335
	CD163	0.156	0.030
*Dendritic Cells (DCs)*	ITGAX	0.072	0.320
	FSCN1	−0.156	0.030
	ADAM19	0.127	0.078
*Natural Killer cells (NK)*	NCR1	0.184	0.010
	NCAM1	−0.096	0.183
	KLRB1	0.108	0.136
*Neutrophils*	CEACAM3	0.033	0.64
	FCGR3B	0.137	0.056
	FPR1	0.105	0.145
*Monocyte*	CSF1R	0.095	0.186
	CD86	0.167	0.020
	C3AR1	0.161	0.025
*Cytokines*	IL2	0.083	0.249
	IL1A	0.182	0.011
	IL10	0.159	0.027
	IL19	0.224	0.001
	IL20	0.196	0.006
	IL21	0.169	0.018
	TNF	0.139	0.053
	CXCR2	0.302	2.14E-05
	CSF1	0.166	0.021
	CD274	0.260	0.0002
	CTLA4	0.171	0.017
	PDCD1	0.169	0.019

**Table 3:** The ACE2 correlated immune cell markers of toll-like receptors, macrophages, dendritic cells, natural killer cells, neutrophils, monocytes and cytokines in HER2 subtype of breast cancer.

Cell Type	Gene markers	COR	P-Values
***Toll-like receptors***	TLR4	0.04	0.666
	TLR7	−0.001	0.986
	TLR8	−0.019	0.864
	TLR9	0.008	0.937
***Macrophage***	MS4A4A	0.065	0.559
	CD68	0.140	0.208
	CD163	0.072	0.515
***Dendritic Cells (DCs)***	ITGAX	0.038	0.730
	FSCN1	−0.110	0.324
	ADAM19	−0.106	0.342
***Natural Killer cells (NK)***	NCR1	0.011	0.915
	NCAM1	−0.227	0.039
	KLRB1	0.014	0.898
***Neutrophils***	CEACAM3	0.152	0.170
	FCGR3B	0.242	0.027
	FPR1	−0.066	0.550
***Monocyte***	CSF1R	0.015	0.891
	CD86	0.015	0.887
	C3AR1	0.020	0.854
***Cytokines***	IL2	−0.09	0.403
	IL1A	0.139	0.211
	IL10	−0.121	0.276
	IL19	−0.08	0.432
	IL20	−0.004	0.970
	IL21	−0.110	0.324
	TNF	−0.10	0.357
	CXCR2	−0.02	0.796
	CSF1	−0.055	0.621
	CD274	0.144	0.194
	CTLA4	0.088	0.431
	PDCD1	0.022	0.837

**Table 4:** The ACE2 correlated immune cell markers of toll-like receptors, macrophages, dendritic cells, natural killer cells, neutrophils, monocytes and cytokines in Luminal B subtype of BC.

Cell Type	Gene markers	COR	P-values
***Toll-like receptors***	TLR4	0.103	0.127
	TLR7	0.045	0.506
	TLR8	0.107	0.113
	TLR9	0.042	0.530
***Macrophage***	MS4A4A	0.056	0.407
	CD68	0.038	0.566
	CD163	0.056	0.407
***Dendritic Cells (DCs)***	ITGAX	0.016	0.807
	FSCN1	0.001	0.987
	ADAM19	0.181	0.007
***Natural Killer cells (NK)***	NCR1	0.154	0.223
	NCAM1	0.188	0.005
	KLRB1	0.140	0.037
***Neutrophils***	CEACAM3	0.095	0.160
	FCGR3B	0.035	0.598
	FPR1	0.057	0.393
***Monocyte***	CSF1R	−0.022	0.742
	CD86	0.095	0.157
	C3AR1	0.067	0.318
***Cytokines***	IL2	0.108	0.108
	IL1A	0.127	0.058
	IL10	0.139	0.039
	IL19	0.077	0.252
	IL20	−0.035	0.603
	IL21	0.187	0.005
	TNF	0.033	0.619
	CXCR2	0.078	0.249
	CSF1	0.009	0.884
	CD274	0.250	0.0001
	CTLA4	0.206	0.071
	PDCD1	0.133	0.048

## 3 Results

### 3.1 UCSC Xena analysis showed low expression of ACE2 in breast invasive carcinoma

**Fig. 1:**
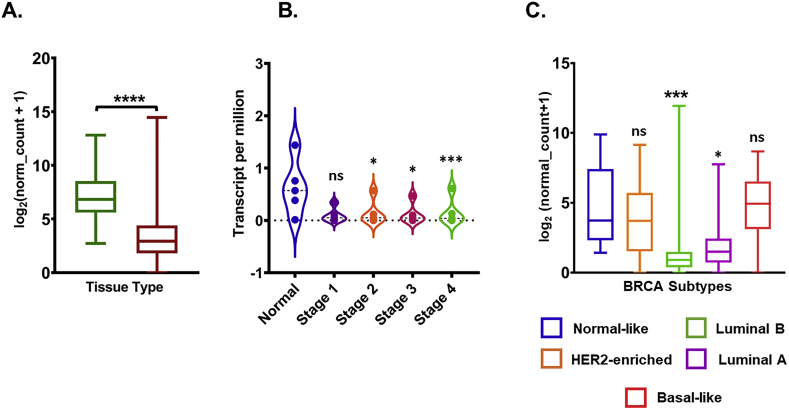
**Gene expression level of ACE2 in breast cancer. (A)** Box plots show the ACE2 mRNA expression in normal and tumor tissue Analysis was done with the UCSC Xena browser using TCGA TARGET GTEx study. **(B)** TCGA Breast cancer (BRCA) study was used to investigate the ACE2 in expression in distinct BRCA molecular subtypes. GraphPad Prism 8.4.2 was used to calculate statistical differences by unpaired *t*-test with Welch's correction. **(C)** Violin plot showing ACE 2 expression levels in BRCA tumor based on tumor stage via UALCAN database. (*P < 0.05, **P < 0.01, ***P < 0.001, ****P < 0.0001).

To understand the transcriptional expression pattern of ACE2 in breast cancer, we analyzed Its mRNA expression levels in normal tissue and tumor tissue using TCGA TARGET GTEx study (n = 1278) which allows one to compare the expression using the TCGA database (tumor tissue) and GTEx database (normal tissue) in cancer patients by using UCSC Xena browser. Analyzed results suggested that the ACE2 mRNA level in breast cancer tumor tissue was significantly lower than that in normal tissue (*P* < 0.001; Fig. 1A). We further analyzed the ACE2 expression with the tumor stage for breast cancer patients in the TCGA database using the UALCAN portal. Consistent with the observations obtained in Fig. 1A, ACE2 expression in Stage 2, 3, and 4 were significantly decreased with normal, stage 1, did not significantly differ (Fig. 1B). As molecular subtypes of breast cancer demonstrate its biological diversity [1], therefore we explore the ACE2 mRNA expression in these subtypes. Obtained results demonstrated that ACE2 mRNA expression was found to be downregulated in luminal (luminal A and B) breast cancer subtypes (Fig. 1C).

### 3.2 ACE2 correlation with various immune cells and their markers

**Fig. 2:**
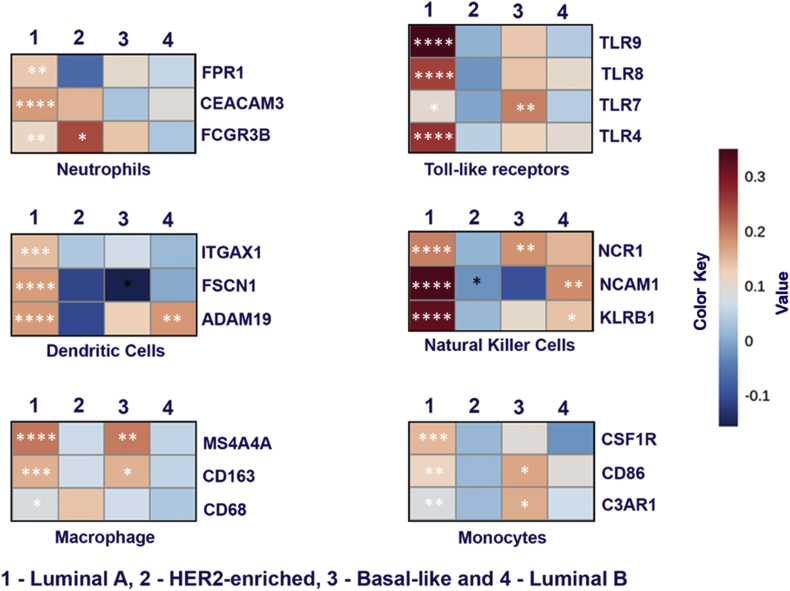
**ACE2 expression correlated with immune cell markers in BC subtypes.** Heat map showing the Spearman correlation-based analysis with the significance of immune cell-specific markers in subtypes of breast cancer using TIMER 2.0 analysis. The markers include FPR1, CEACAM3 and FCGR3B of neutrophils, TLR4, TLR7, TLR8 and TLR9 of toll-like receptors, ITGAX1, FSCN1 and ADAM19 of dendritic cells, NCR1, NCAM1 and KLRB1 of natural killer cells, MS4A4A, CD163 and CD68 of macrophages and CSF1R, CD86 and C3AR1 of monocyte cells.

To investigate the correlation of ACE2 expression and TIL in breast cancer, we focused on some of the major markers of various immune cell types. TIMER 2.0 database was used to analyze the correlation of ACE2 with different immune cell type markers such as Toll-like receptors (TLR's), dendritic cells (DC), macrophages, monocytes, neutrophils, and natural killer (NK) cells. Heat-map was generated based on the correlation values obtained between these markers and ACE2 expression (Fig. 2). Obtained results suggested that ACE2 in luminal A subtype was significantly positively correlated with all the major markers like TLRs (TLR4, TLR7, TLR8, and TLR9), NK cells (NCR1, NCAM1, and KLRB1), Neutrophils (CEACAM3, FCGR3B, and FPR1), Monocytes (CSF1R, CD86, and C3AR1), Macrophage (MS4A4A, CD68, and CD163) and Dendritic Cells (ITGAX, FSCN1, and ADAM19) indicating immunological susceptibility of luminal A subtype cancer towards ACE2. Among other Her-2, Basal and luminal B subtypes, in basal subtypes significant positive correlation for TLR7, MS4A4A CD163, NCR1, and CD86 gene markers (Table 2). In Her-2, the FCGR3B gene marker (Table 3) and in luminal B, NCAM1, and KLRB1 gene markers (Table 4) are significantly positively correlated. We also analyzed the correlation of immune cells in ACE2 function such as CD4^+^, CD8^+^ and B cells in breast cancer subtypes. CD4^+^ and CD8^+^ cells exhibited the significantly positive correlation in luminal A and luminal B subtype (Supplementary Fig. 5). The results were further strengthening our hypothesis that luminal A and luminal B subtypes are crucial to the role of ACE2. However, B cell was not found to be correlated to the ACE2. As TMPRSS2, a transmembrane serine protease is involved in entry of SARS-CoV2, we analyzed the expression of TMPRSS2 correlated with immune cell infiltrations. Neutrophils and dendritic cells (DC) exhibited the significantly positive correlation in luminal A subtype only (Supplementary Fig. 6). This indicates the role of TMPRSS2 in luminal A subtype through TIL.

### 3.3 Cytokines modulation in correlation with ACE2 in breast cancer subtypes

**Fig. 3:**
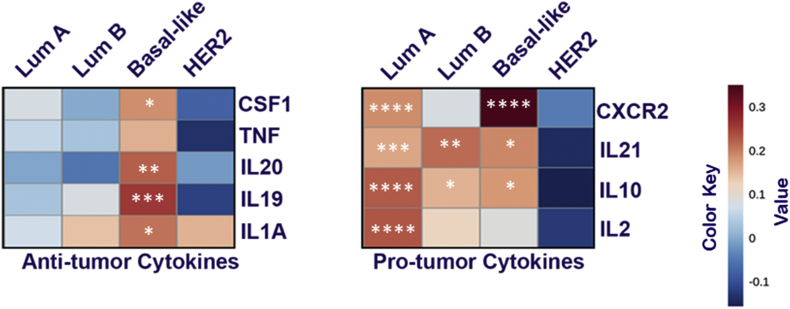
**ACE2 expression correlated with cell markers of “pro-tumorigenic” and “anti-tumorigenic” in breast cancer subtypes.** Heat map showing the Spearman correlation-based analysis with the significance of cytokines specific markers in subtypes of BC using TMER 2.0 analysis. Markers include CSF1, TNF, IL20, IL19, and IL1A of anti-tumor cytokines and CXCR2, IL21, IL10, and IL2 of pro-tumor cytokines.

The term “cytokine storm” has become increasingly used not in the case of COVID-19, which is the main cause of Acute Respiratory Distress Syndrome (ARDS) [33]. We analyzed ACE-2 dependent cytokine modulation in breast cancer subtypes in context with its Pro or Anti-tumorigenic cytokines modulation. Heat-map was generated based on the correlation (Table 1, Table 2, Table 3, Table 4) between pro/anti-tumorigenic cytokines gene and ACE2 expression is shown in Fig. 3. Obtained results suggested that in luminal A subtype, ACE2 was significantly (*P* > 0.05) positively correlated with anti-tumorigenic cytokines IL-2, IL-10, IL-21, and CXCR2 and negatively correlated with protumorigenic cytokines such as IL-1A, IL-19, IL-20, TNF, and CSF1. Similar results were obtained in the case of the luminal B subtype, except only one pro-tumorigenic cytokine was positively correlated with ACE2. Surprisingly, in the basal subtype, we found a positive correlation of both Pro and Anti-tumorigenic cytokines, on the contrary Her-2 enrich breast cancer subtype was negatively correlated for both types of cytokines.

**Fig. 4:**
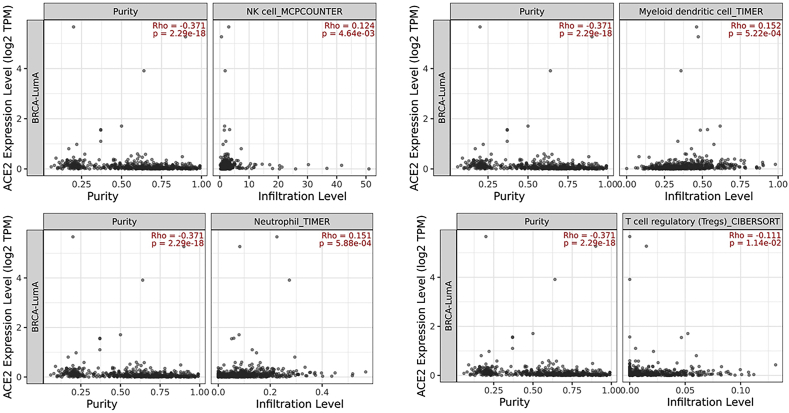
**ACE2 expression correlated with immune cells in breast cancer in BC subtypes.** The correlation of ACE2 mRNA expression with tumor-infiltrating neutrophils, dendritic cells, macrophages, and T regulatory (Treg) cells in BC subtypes based on TIMER 2.0 database analysis.

To further explore the extent of immune cell infiltration in breast cancer, the association between immune cells and ACE2 was evaluated using TIMER 2.0. Similarly, ACE2 was found to be significantly correlated with neutrophils (Supplementary Fig. 1), dendritic cells (DC) (Supplementary Fig. 2), T cell regulatory (Tregs) (Supplementary Fig. 3) and natural killer cells (NK) Supplementary Fig. 4) cells which are reported to play a protective role in host defense against luminal A subtype (Fig. 4). Evidence suggests that regulatory T cells (Tregs) correlated with worse outcome in breast cancer [34,35]. Our results also show the negative correlation of ACE2 with Tregs which further confirms the protective role of ACE2 in luminal A breast cancer subtype (Fig. 4).

## 4 Discussion

In the present study, we identified that the transcription signature of the ACE2 gene is varying in various molecular subtypes of breast cancer. ACE2 mRNA expression is significantly downregulated in luminal A and luminal B subtypes when compared to other subtypes (HER-2 enrich and Basal subtype) as well as in normal breast tissue. Downregulation of ACE2 is associated with worst prognosis in breast cancer [36], however, how various molecular breast cancer subtypes vary with ACE2 mRNA expression was still unexplored. Here, we identified that ACE2 mRNA expression was significantly down-regulated in stage II, III, and IV of breast cancer compared with stage I, indicating that the decrease in ACE2 expression may play a vital role in the development and differentiation of breast cancer.

**Fig. 5:**
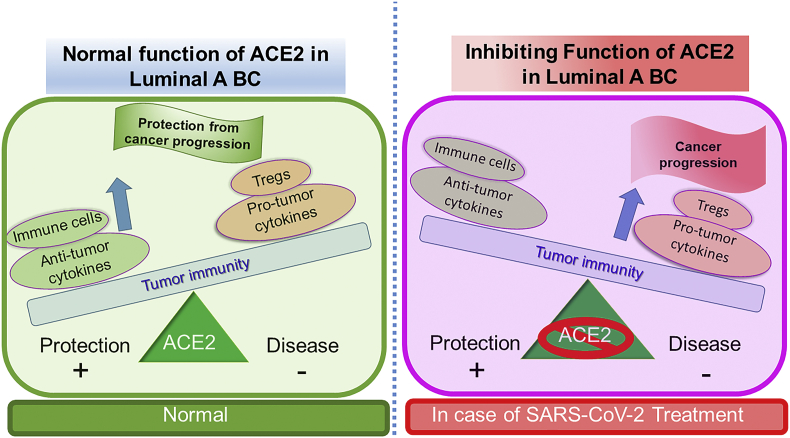
***“The immune balance”***. Predicted hypothese that in luminial A subtype inhibition of ACE2 might lead to disturbed immune balance which favour the tumor growth. In this figure, the balance has shifted towards disease state in ACE2 inhibition condition which can be correlated with a treatment option for current pandemic, COVID-19. Increase of Pro-tumogenic cytokines and Tregs outweigh the anti-tumorogenic cytokine and important immune cells. This would give a good chance for the naive tumor to grow, develop and metastasize.

Genetic or epigenetic mechanisms are primarily involved in the down-regulation of gene expression in various types of cancers [37]. The tumor suppressor function of ACE2 has been investigated in lung cancer [38,39], pancreatic cancer [40], Colon cancer [41] including breast cancer [36] however the cellular consequences of its silencing in different breast cancer subtypes remain to be investigated. To explore the cellular consequences of low expression in luminal A and luminal B breast cancer subtypes, we analyzed the TCGA database using the TIMER 2.0 database. Analyzed data of correlation between ACE2 and TIL in breast cancer subtypes demonstrated that all the major immune cell markers of dendritic cell, macrophages, monocytes, neutrophils, NK cells, and Toll-like receptor in luminal A subtypes are significantly positive correlated (Fig. 2). The luminal A subtype is associated with a low immunological response [42], however our results highlighted positive correlation of ACE2 in TIL which underlying crucial immunological role of ACE2 in breast cancer, leading to poor prognosis as presence of TILs is associated poor prognosis in luminal subtypes [43]. This association is further followed by other breast cancer subtypes such as Basal and Luminal B subtypes with only few immune markers are positively correlated (Fig. 2). To understand the significance of these results in context with the current COVID-19 pandemic, we explore the various treatment options currently in use for the treatment of this disease. ACE2 receptor has been an entry point for the SARS-CoV-2 virus, therefore ACEIs are considered prime candidates for the management of this outbreak [44]. The controversy about the severity of COVID-19 is increased with the patients with comorbidities, which include cardiovascular disease, diabetes, and cancer leading to the worst outcome [45] continues unchanged, with new interpretations about this appearing almost daily. Our analysis strongly support the positive involvement of ACE2 in pro- and anti-tumor cytokines (Fig. 3) and modulation of immune cells (Fig. 4) in the case of luminal A subtype predicting a strong rationale for the consequence of ACEIs in promoting breast cancer progression (Fig. 5). More surprisingly in luminal, A subtype ACE2 is negatively correlated with regulatory T cells (Tregs), which regulate the host responses to infection and neoplasms [46]. Moreover, tumor cells use Tregs as a shield to protect themselves against anti-tumor immune response [46], therefore we propose that blocking ACE2 in luminal A subtype will promote cancer progression (Fig. 5).

Mounting evidence suggested that cytokine strom is one of the prime factor for therapeutic unpredictability and severity of the disease leading to death [47]. As ACE2 is entry point for SARS-CoV-2, therefore inhibiting these receptor had been shown to lower rate of disease severity in patients treated with ACEIs [48]. In these patients one of the interlukin, IL-6, was found to be lowered in peripheral blood highlighting immunomodulatory role of ACEi. Similarly we also found that various interleuking also significantly corelated in luminal A breast cancer subtypes (Fig. 3). Furthermore, ACEIs also downregulate CD3 and CD8 cell counts which is inversely associated with CD8 T cell functional avidity in HIV-infected people [49]. Still the role ACEIs in immune modulation is much debatable as study reported ACEIs/ARBs prior to COVID‐19 infection could reduce the severity of COVID‐19 [50] which is further contradicted by studies which does not found any significant correlation with ACEIs/ARBs and the severity and mortality of patients with COVID‐19 [51]. Here, we reported that the comparative gene expression of ACE2 each breast cancer subtypes defining its pathological role in different subtypes of breast cancer. Our analysis suggested expression of ACE2 gene corelated with immune function.

This study is much signficant in context with current pandemic in which ACE2 inhibitor are widely used for the COVD-19 treatment. Finally, we concluded that ACEIs treatment in Luminal A breast cancer may promote tumor progression however futher research is needed to validate these finding and to investigate new means of COVID-19 treatment.

## Author contributions statement

**Rajeev Mishra**: Supervision, Conceptualization, Methodology, Software, Writing- Reviewing and Editing **Vikas Kumar Bhari**: Conceptualization, Methodology, Software, Data curation, Writing- Original draft preparation. **Durgesh Kumar, Surendra Kumar**: Conceptualization,Writing- Reviewing and Editing.

## Declaration of competing interest

The authors declare that they have no known competing financial interests or personal relationships that could have appeared to influence the work reported in this paper.
