# Supplementary material for: SARS-CoV-2 cell receptor gene ACE2 -mediated immunomodulation in breast cancer subtypes
Source: Biochem Biophys Rep. 2020 Nov 5;24:100844. doi: 10.1016/j.bbrep.2020.100844 (PMC7643628; doi:10.1016/j.bbrep.2020.100844)

**Supplementary Figure 1** – Correlation of ACE2 expression with immune infiltration of neutrophils in breast cancer subtypes.

**Neutrophils –**

**Luminal B** **HER2**


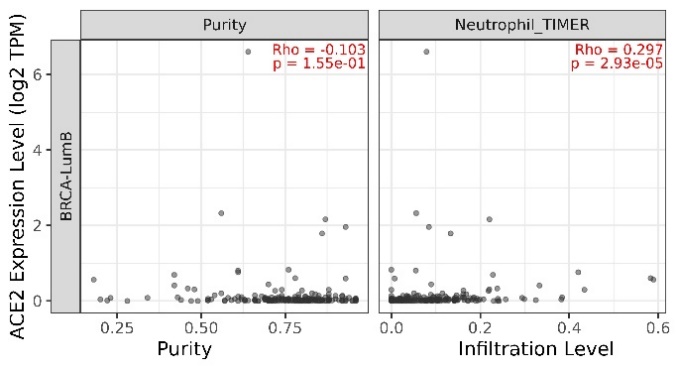

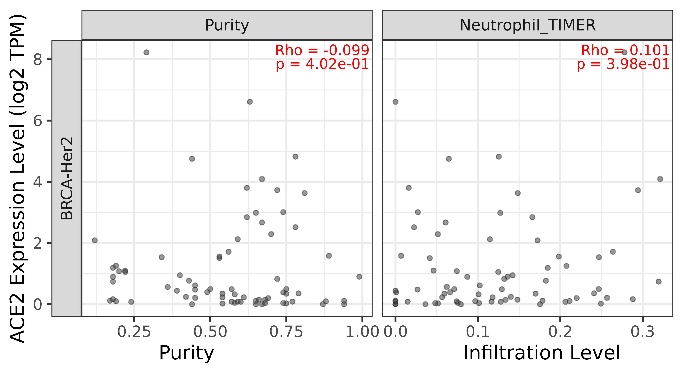


**Basal-like**


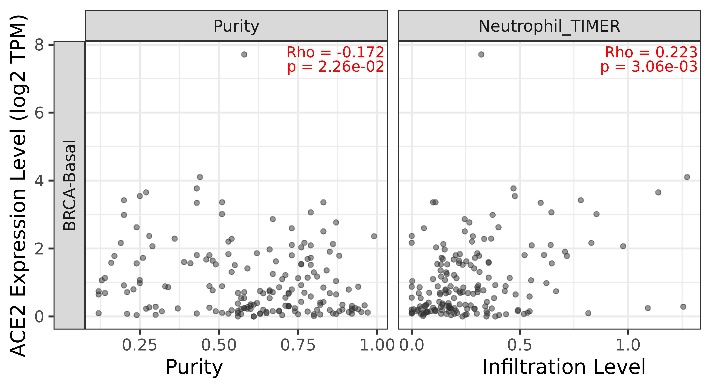


**Supplementary Figure 2** – Correlation of ACE2 expression with immune infiltration of Dendritic cell in breast cancer subtypes.

**Dendritic cells –**

**Luminal B** **HER2**


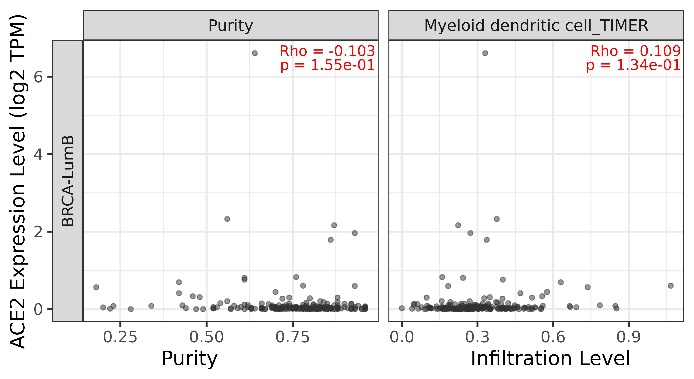

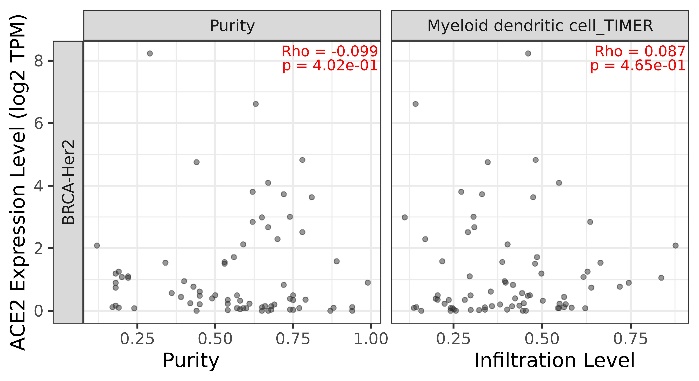


**Basal-like**


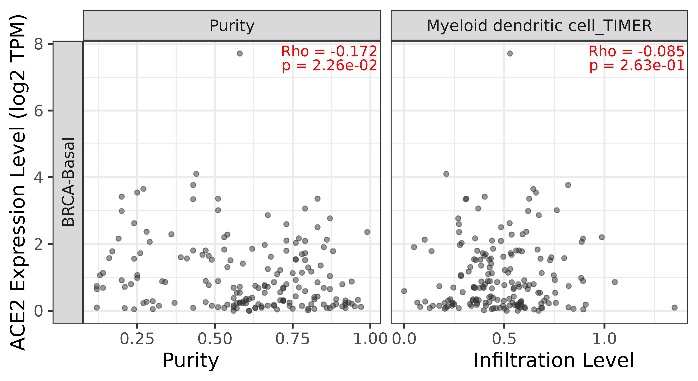


**Supplementary Figure 3** – Correlation of ACE2 expression with immune infiltration of T cell regulatory (Tregs) in breast cancer subtypes.

**T cell regulatory (Tregs) –**

**Luminal B** **HER2**


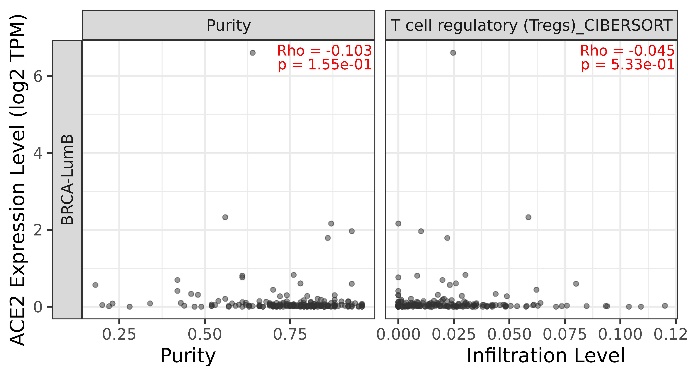

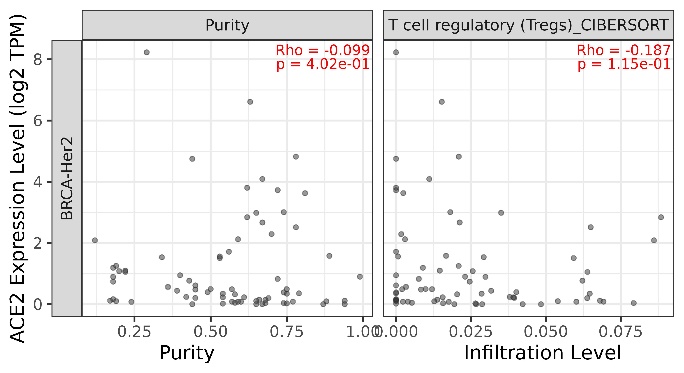


**Basal-like**


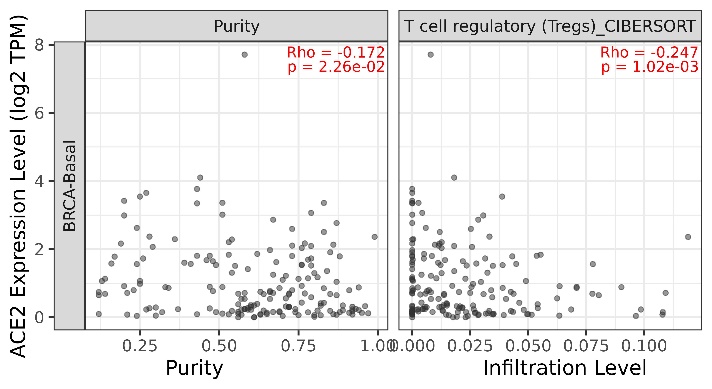


**Supplementary Figure 4** – Correlation of ACE2 expression with immune infiltration of natural killer cells in breast cancer subtypes.

**Natural Killer cells (NK) –**

**Luminal B** **HER2**


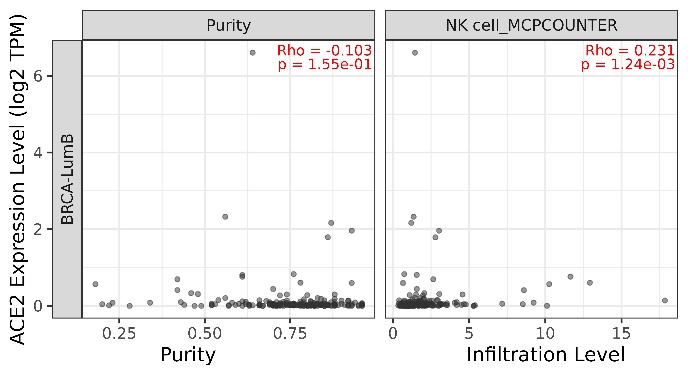

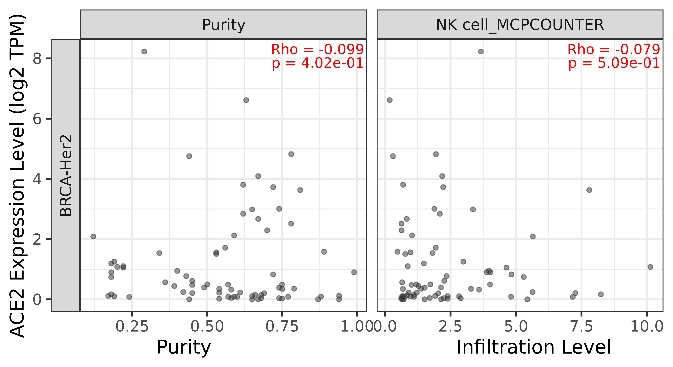


**Basal-like**


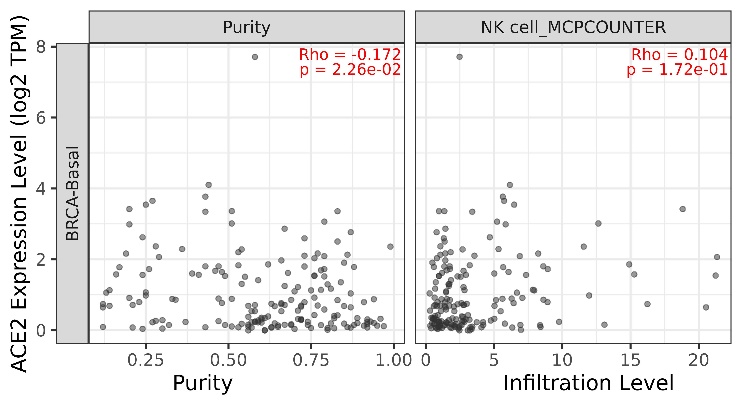


**Supplementary Figure 5 : The ACE2 correlated CD4+ T cells, CD8+ T cells and B cell infiltration in breast cancer subtypes.** ACE2 expression exhibit significant positive correlation with CD4+ T cells and CD8+ T cells in luminal A and luminal B subtype while B cells do not show any significance in any cancer subtype.


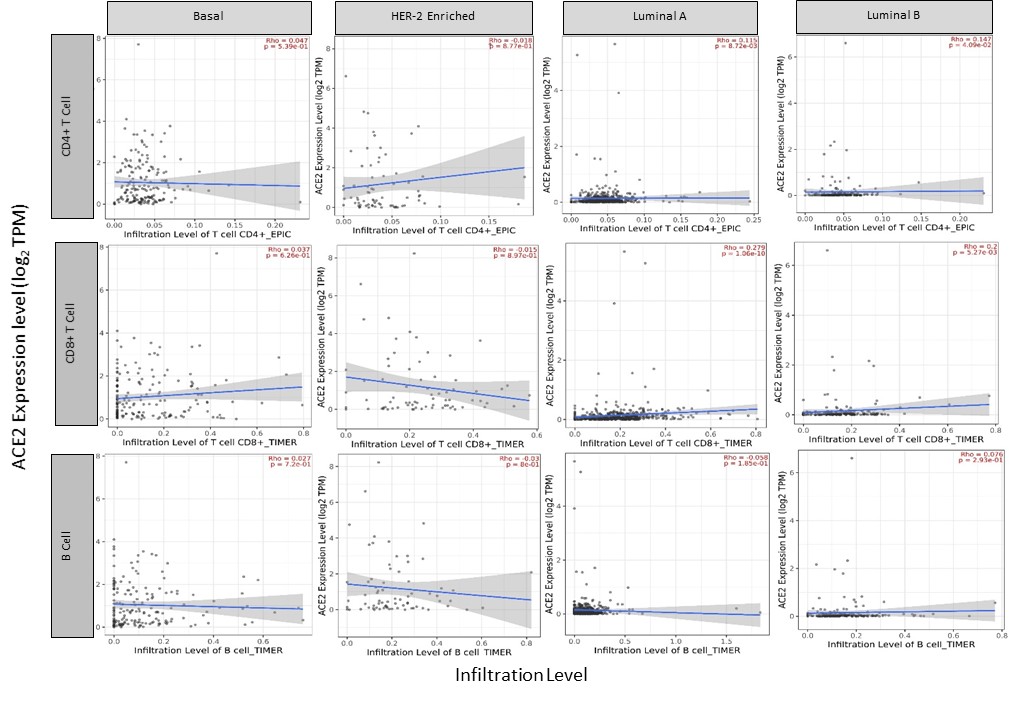


**Supplementary Figure 6 : The TMPRSS2 correlated neutrophil and Dendritic cells (DC) infiltration in breast cancer subtypes.** TMPRSS2 expression exhibit significant positive correlation with neutrophil and DC cells in luminal A and luminal B subtype.


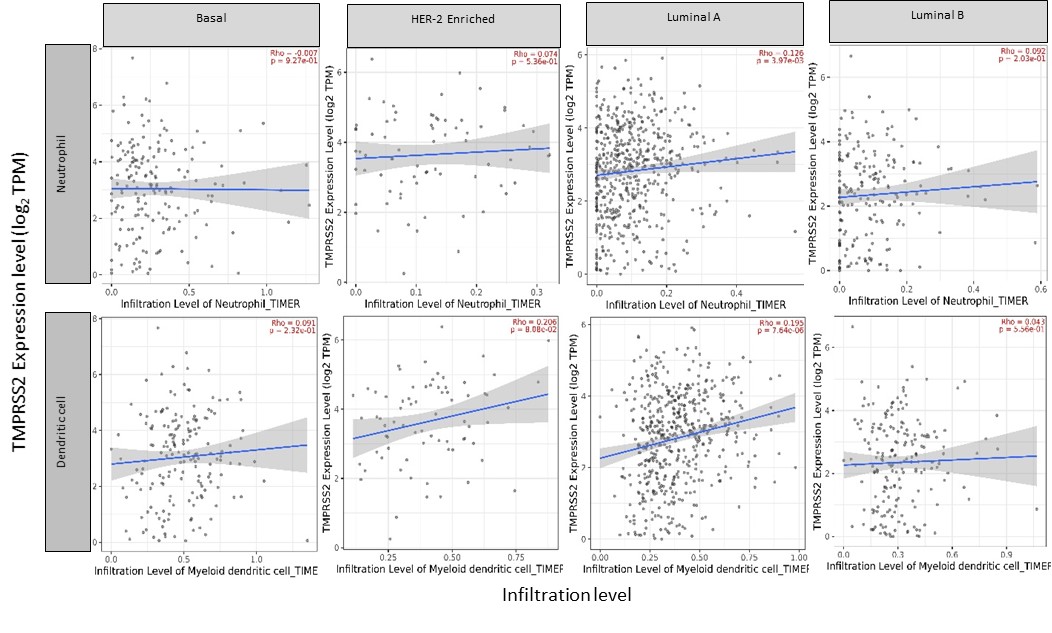

Supplement: Multimedia component 1 [file mmc1.doc]
